# A Multi-Omics Analysis of NASH-Related Prognostic Biomarkers Associated with Drug Sensitivity and Immune Infiltration in Hepatocellular Carcinoma

**DOI:** 10.3390/jcm12041286

**Published:** 2023-02-06

**Authors:** Yongting Liu, Zhaohui Jiang, Xin Zhou, Yin Li, Ping Liu, Yihong Chen, Jun Tan, Changjing Cai, Ying Han, Shan Zeng, Hong Shen, Ziyang Feng

**Affiliations:** 1 Department of Oncology, Xiangya Hospital, Central South University, Changsha 410008, China; 2 Department of Neurosurgery, Xiangya Hospital, Central South University, Changsha 410008, China; 3 National Clinical Research Center for Geriatric Disorders, Xiangya Hospital, Central South University, Changsha 410008, China

**Keywords:** multi-omics analysis, NASH-driven HCC, prognostic biomarkers, drug sensitivity, immune infiltration

## Abstract

**Background**: Nonalcoholic steatohepatitis (NASH)-driven hepatocellular carcinoma (HCC) is becoming a major health-related problem. The exploration of NASH-related prognostic biomarkers and therapeutic targets is necessary. **Methods**: Data were downloaded from the GEO database. The “glmnet” package was used to identify differentially expressed genes (DEGs). The prognostic model was constructed by the univariate Cox and LASSO regression analyses. Validation of the expression and prognosis by immunohistochemistry (IHC) in vitro. Drug sensitivity and immune cell infiltration were analyzed by CTR-DB and ImmuCellAI. **Results**: We constructed a prognostic model that identified the NASH-related gene set (DLAT, IDH3B, and MAP3K4), which was validated in a real-world cohort. Next, seven prognostic transcription factors (TFs) were identified. The prognostic ceRNA network included three mRNAs, four miRNAs, and seven lncRNAs. Finally, we found that the gene set was associated with drug response which was validated in six clinical trial cohorts. Moreover, the expression level of the gene set was inversely correlated with CD8 T cell infiltration in HCC. **Conclusions**: We established a NASH-related prognostic model. Upstream transcriptome analysis and the ceRNA network provided clues for mechanism exploration. The mutant profile, drug sensitivity, and immune infiltration analysis further guided precise diagnosis and treatment strategies.

## 1. Introduction

Hepatocellular carcinoma (HCC) is one of the most common cancers with an increasing mortality rate [1,2]. Nonalcoholic steatohepatitis (NASH), a manifestation of systemic metabolic disease, has been recognized as a leading cause of HCC and liver transplantation in recent years [3]. Although the incidence of NASH-driven HCC is lower than that of other causes (such as hepatitis virus), the number of NASH patients is large, and the incidence of NASH is projected to double within 10 years [4].

Compared to hepatitis virus-driven HCC, up to 50% of NASH-driven HCC occurs in patients without cirrhosis, with relatively low annual incidence [3]. These features lead to delays in diagnosing and treating NASH-driven HCC, ultimately affecting cancer prognosis. With the development of high-throughput sequencing and bioinformatics, a range of potential biomarkers that play a role in HCV-, HDV-, type 2 diabetes mellitus-, and metallothionein1 deletion-driven HCC has been identified [5,6,7,8]. Some pyroptosis-related genes (GSDMC, DHX9, CHMP4B, BAK1, and NOD2), necroptosis-related genes (RIPK1, RIPK3, and MLKL-p), anoikis-related genes (DAP3), and cuproptosis-related genes (TAF6, SPP2, CFHR4, DNASE1L3) have also been defined as prognostic targets for HCC [9,10,11,12]. A systematic understanding of disease pathogenesis and treatment response may help develop new approaches to cancer diagnosis and treatment. Therefore, more perspectives are needed to identify prognostic biomarkers and underlying mechanisms associated with NASH-driven HCC.

In this study, we aimed to establish a risk model based on NASH-related differentially expressed genes (DEGs) that can predict the prognosis of HCC. Furthermore, upstream regulatory networks, mutational profiles, drug sensitivity, and immune infiltration were studied by multi-omics analysis.

## 2. Materials and Methods

### 2.1. Differential Expression Analysis

Download the data of GSE89632 (NASH), GSE99807 (NASH-driven HCC), and GSE87630 (HCC) from the online tool GEO2R (https://www.ncbi.nlm.nih.gov/geo/geo2r accessed on 10 October 2022). The 3 databases had complete clinical information and sufficient samples for analysis. DEGs were obtained through “limma” packages [13]. |log2FC| > 1 and adj. *p*-value < 0.05 were set as the cut-off criteria. Protein–protein interactions (PPIs) were obtained from the STRING database (https://string-db.org/ accessed on 10 October 2022).

### 2.2. Functional Enrichment Analysis

Gene Ontology (GO) and Kyoto Encyclopedia of Genes and Genomes (KEGG) analysis were carried out through R software, including the “clusterProfiler”, “enrichplot”, and “goplot” packages. GO analysis includes three terms: biological process (BP), molecular function (MF), and cellular component (CC). The top 5 enriched GO terms and KEGG pathways were displayed using the “ggplot2” package.

### 2.3. Construction of a Prognostic Model and Nomogram

The least absolute shrinkage and selection operator (LASSO) regression model was performed using the “glmnet” package in TCGA-LIHC. Univariate Cox regression analysis further screened the DEGs with prognostic value using the “survival” package. The risk score was calculated according to the centralized and standardized mRNA expression data in TCGA-LIHC. $\mathrm{Risk}\;\mathrm{score}=\sum_{i=1}^{n}\exp\left(\mathrm{Xi}\right)\;\mathrm{coef}\;\left(\mathrm{Xi}\right).$ Here, risk score = expression level of DLAT × 0.255685 + expression level of IDH3B × 0.027537 + expression level of MAP3K4 × 0.190317. Next, the overall survival (OS) curves and receiver operating characteristic (ROC) curves were produced by the “survival” package and “timeROC” package, respectively. The “rms” and “survival” packages were used to form nomogram and calibration plots.

### 2.4. Specimen Collection

Liver tissues were obtained from Xiangya hospital. Two highly qualified pathologists independently diagnosed these samples. HCC tissues and their adjacent normal tissues were collected and immediately transferred to −80 °C for storage once excised. Formalin-fixed tissue samples were embedded with paraffin for subsequent immunohistochemistry (IHC). This study was performed in accordance with the Declaration of Helsinki and approved by the Ethics Committee of Xiangya hospital (ID: 202105066). All participants/patients have given informed consent.

### 2.5. Immunohistochemistry (IHC)

After dehydration and paraffin embedding, the liver tissues fixed with 4% paraformaldehyde were prepared into tissue chips. After dewaxing and hydration, 10 mM sodium citrate was used at 95 °C for 15 min for antigen repair. After cooling to room temperature, endogenous peroxidase was blocked with 3% H2O2 for 10 min. The diluted primary antibodies (DLAT Afantibody Cat# AF302309; IDH3B Afantibody Cat# AF10574; MAP3K4 Afantibody Cat# AF05249) were then incubated at 4℃ overnight. Next, biotin-labeled IgG (Abcam Cat# ab6721) was used to detect antibody binding. DAB-H_2_O_2_ and hematoxylin were prepared for staining. Images were photographed with a microscope. The staining score was defined by both the staining intensity score and the positive staining percentage score. The staining intensity was divided into 4 grades: negative is 0; weakly positive is 1; moderately positive is 2; strongly positive is 3. The percentage of positive staining was divided into 4 groups: 1 means positive in 1–25%; 2 means positive in 26–50%; 3 means positive in 51–75%; 4 means positive in 76–100%.

### 2.6. TF-Gene Interaction and Competing Endogenous RNA (ceRNA) Network

Transcription factors (TFs) were predicted at the single-cell or bulk level through GRNdb (http://www.grndb.com/ accessed on 10 October 2022) [14]. miRNAs and lncRNA were predicted based on mirDIP (http://ophid.utoronto.ca/mirDIP/ accessed on 10 October 2022) [15] and StarBase V2.0 (https://starbase.sysu.edu.cn/ accessed on 10 October 2022) [16]. The network diagrams were made by the “igraph” package.

### 2.7. Mutation Analysis

The single-nucleotide variant (SNV) and copy number variation (CNV) data were collected from the TCGA database. Seven types of mutation were included in this analysis: Missense_Mutation, Nonsense_Mutation, Frame_Shift_Ins, Splice_Site, Frame_Shift_Del, In_Frame_Del, and In_Frame_Ins. These types of mutations are deleterious mutations. Moreover, the non-deleterious mutations include Silent, Intron, IGR, 3’UTR, 5’UTR, 3’Flank, and 5’Flank. The copy number variation (CNV) data were processed through GISTIC2.0 [17].

### 2.8. Drug Sensitivity and Response Analysis

We collected the IC50 data and mRNA gene expression from the Genomics of Therapeutics Response Portal (GTRP) (https://portals.broadinstitute.org/ accessed on 10 October 2022) [18]. Pearson correlation analysis was performed to obtain the correlation between gene mRNA expression and drug IC50. Data on drug response to cancer treatments were obtained from the cancer treatment response gene signature database (CTR-DB) (http://ctrdb.cloudna.cn/ accessed on 10 October 2022) [19]. CTR_Microarray_14, CTR_Microarray_15, CTR_Microarray_35, CTR_Microarray_71, CTR_RNAseq_410, and CTR_RNAseq_386 were analyzed. The ability to predict drug response was based on the AUC value.

### 2.9. Immune Cell Abundance Identifier (ImmuCellAI)

The association between gene expression and immune cell infiltration was analyzed by ImmuCellAI (https://github.com/lydiaMyr/ImmuCellAI accessed on 10 October 2022) [20]. Spearman’s coefficient was used for evaluation. The immune infiltration data were obtained from TCGA. Heatmaps were drawn through the “ggplot2” package.

### 2.10. Statistical Analysis

Statistical analysis was performed in R software (v.3.6.3) and GraphPad Prism. The differential expression analyses were detected with *t*-test. The survival analyses were determined by the Logrank test. In all analyses, *p*-value < 0.05 was considered statistically significant. *, **, and *** indicated *p* < 0.05, *p* < 0.01, and *p* < 0.001, respectively.

## 3. Results

### 3.1. Screening of the Common DEGs in NASH and HCC

**Figure 1:**
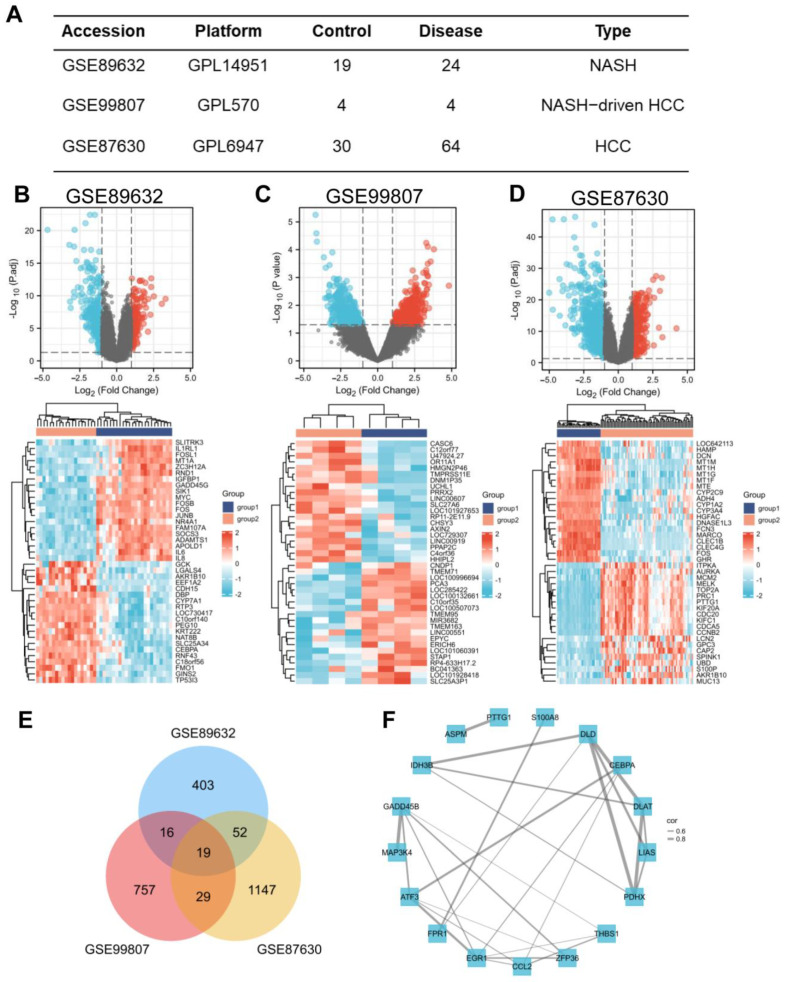
Screening of the common DEGs in NASH and HCC. (**A**) Description of datasets used to screen DEGs. (**B**−**D**) The volcano plots (|log2(FC)| > 1, adj. *p* < 0.05) and heatmap plots (top 20) of DEGs in GSE89632, GSE99807, and GSE87630. (**E**) Overlap of DEGs from 3 datasets found 19 valuable genes. (**F**) Protein–protein interaction network of 19 DEGs among NASH and HCC. The width of the edge represents the correlation.

In search of gene expression microarrays regarding NASH and HCC in the GEO database, three datasets (GSE89632, GSE99807, and GSE87630) were finally included (Figure 1A). Subsequently, differential expression analysis was conducted. A total of 487 DEGs were obtained in GSE89632, 828 DEGs were obtained in GSE99807, and 1247 DEGs in GSE87630. The corresponding volcano maps and heat maps (top 20) are shown in Figure 1B–D. Next, we further identified 19 DEGs that appeared in all 3 databases (Figure 1E). The gene symbols and interactions of these 19 significant DEGs are presented in Figure 1F and Table S1.

### 3.2. Functional Enrichment Analysis for the Common DEGs

**Figure 2:**
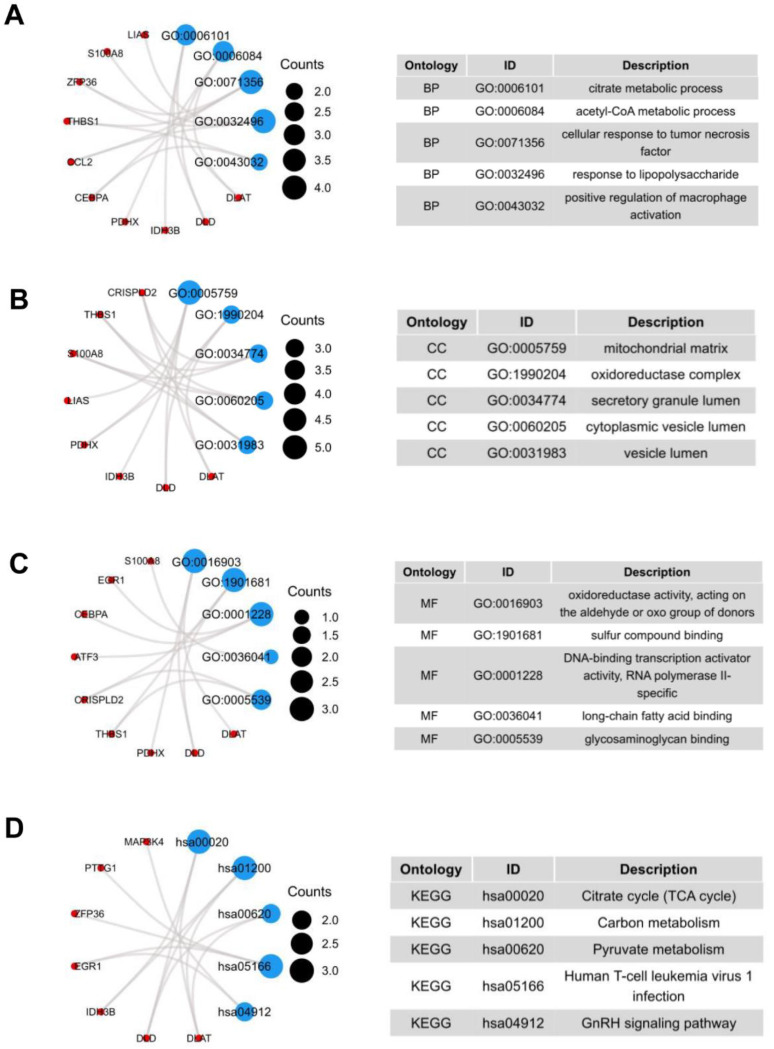
GO terms and KEGG pathway of the common 19 DEGs. (**A**) Biological Processes, (**B**) Cellular Component, (**C**) Molecular Function, (**D**) KEGG Human Pathway.

We performed functional enrichment analysis to predict the underlying biological function and corresponding pathways between NASH and HCC. The enriched GO functions included citrate metabolic process and acetyl-CoA metabolic process in the BP category (Figure 2A); mitochondrial matrix, oxidoreductase complex, and secretory granule lumen in the CC category (Figure 2B); oxidoreductase activity, acting on the aldehyde or oxo group of donors and sulfur compound binding in the MF category (Figure 2C). Some enriched KEGG pathways were also observed, among which citrate cycle, carbon metabolism, and pyruvate metabolism were the most highly enriched pathways (Figure 2D).

### 3.3. Construction and Validation of a Prognostic Model

**Figure 3:**
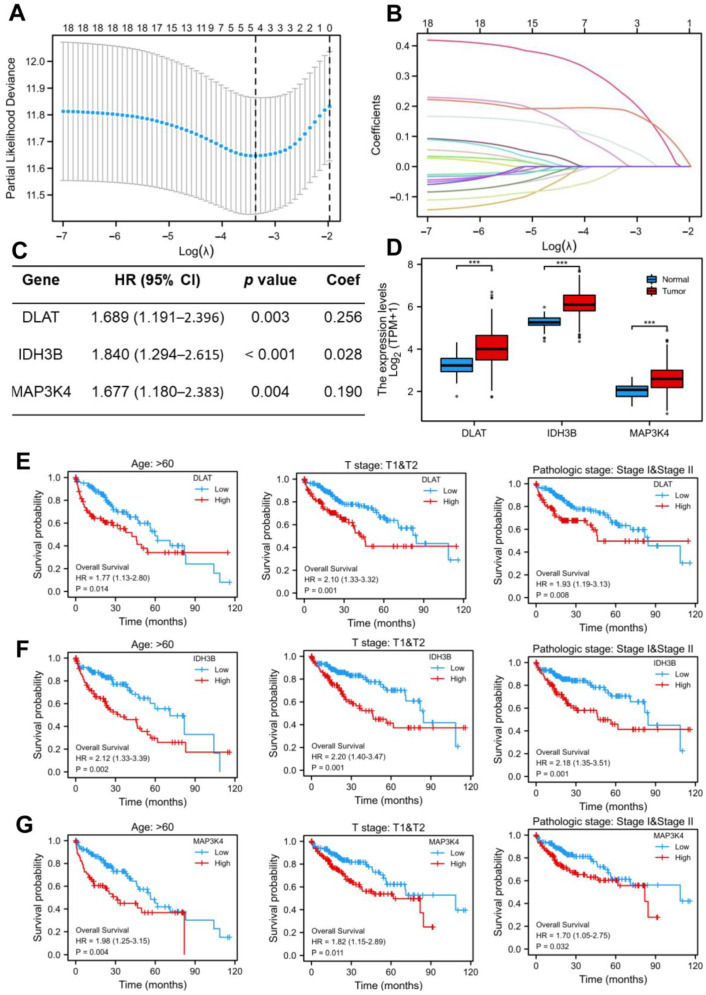
Construction of the LASSO regression model. (**A**) Cross-validation for tuning the parameter selection in the LASSO regression. (**B**) Coefficient profiles in the lasso regression model. The different color lines represented the trajectory of the coefficients for each variable. (**C**) Univariate Cox regression analysis and LASSO coefficient of DLAT, IDH3B, and MAP3K4 in TCGA-LIHC. (**D**) The expression levels of DLAT, IDH3B, and MAP3K4 in TCGA-LIHC. (**E**–**G**) Prognostic value of DLAT, IDH3B, and MAP3K4 in different subgroups: T3 and T4 stage, pathological stage III and IV, and fibrosis Ishak score 3–6.

As shown in Figure 1F, we screened out 19 common DEGs. The LASSO regression algorithm and Cox regression analysis were used to refine the gene set (Figure 3A–C) to reduce the overfitting of high-dimensional genes. In this manner, three genes were identified as the most valuable predictive DEGs, and the risk score system was established using the formula mentioned above. In addition, we also analyzed the expression levels of DLAT, IDH3B, and MAP3K4 in HCC (Figure 3D) and their prognostic value in different clinical subgroups: T3 and T4 stage, pathological stage III and IV, fibrosis Ishak score 3–6, and Child–Pugh grade B and C. The results indicated that the lower expression of the three genes might be correlated with better prognosis in advanced patients (Figure 3E–G, Figure S1).

**Figure 4:**
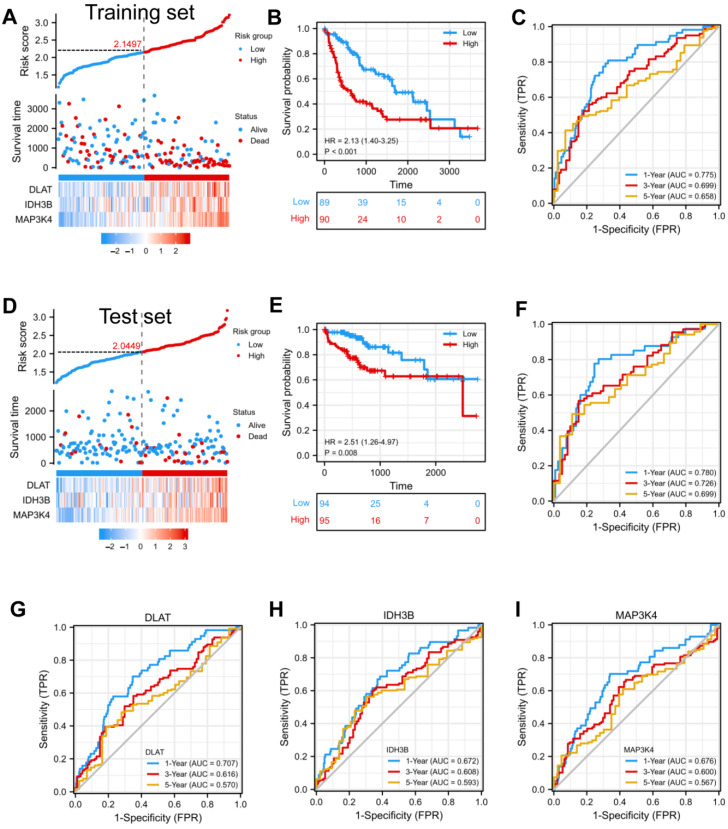
Construction and validation of a prognostic model. (**A**) Risk score, patient survival status, and expression heatmap of 3 DEGs in the training set. (**B**,**C**) Kaplan–Meier curve and time–ROC curve of risk score in the training set. (**D**) Risk score, patient survival status, and expression heatmap of 3 DEGs in the test set. (**E**,**F**) Kaplan–Meier curve and time–ROC curve of risk score in the test set. (**G**–**I**) The predictive efficiency (1, 3, and 5 years) of DLAT, IDH3B, and MAP3K4 in TCGA-LIHC.

In order to confirm whether this risk model could predict the prognosis better, patients from the TCGA-LIHC database were randomly divided into a training set (50%) and a test set (50%). The median risk score was defined as the threshold, dividing the HCC patients into high-risk and low-risk groups. We found that the high-risk group had a higher mortality rate compared with the low-risk group in the training set (Figure 4A). Kaplan–Meier curve showed that patients in the high-risk group had a worse prognosis (*p* < 0.001) (Figure 4B). Moreover, the prognostic accuracy of OS was 0.775 at one year, 0.699 at three years, and 0.658 at five years (Figure 4C). In the test set, the high- and low-risk groups exhibited significantly different survival probabilities (*p* = 0.008) with an AUC of 0.780, 0.726, and 0.699 in a time-dependent ROC at one, three, and five years (Figure 4D–F). In addition, the prognostic model constructed based on our risk score was superior to the single gene (Figure 4G–I) and other models that have been published [21,22,23,24].

### 3.4. Construction of the Nomogram and Calibration Curves

**Figure 5:**
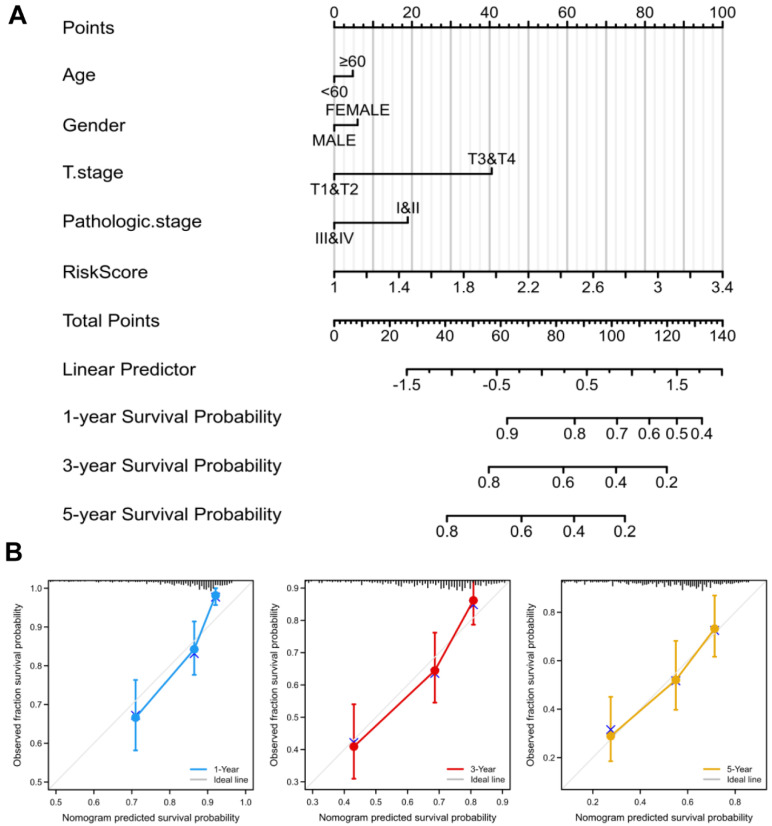
Nomogram to predict survival probability of HCC patients. (**A**) The predictive probability of the risk score with pathologic features in the nomogram. (**B**) Calibration plots for predicting 1-, 3-, and 5-year OS of HCC patients.

To better guide clinical decision-making, we established a nomogram combining multiple clinical parameters, including age, gender, T stage, pathological stage, and risk scores (Figure 5A). In the nomogram, each signature was assigned points according to its risk contribution to OS. Furthermore, we constructed calibration curves, which illustrated a remarkable consensus with the actual survival time of patients with HCC (Figure 5B). Based on these findings, we found that the nomogram containing our risk scores performed well in predicting OS in HCC.

### 3.5. Validation of the Expression and Prognosis in a Real-World HCC Cohort

**Figure 6:**
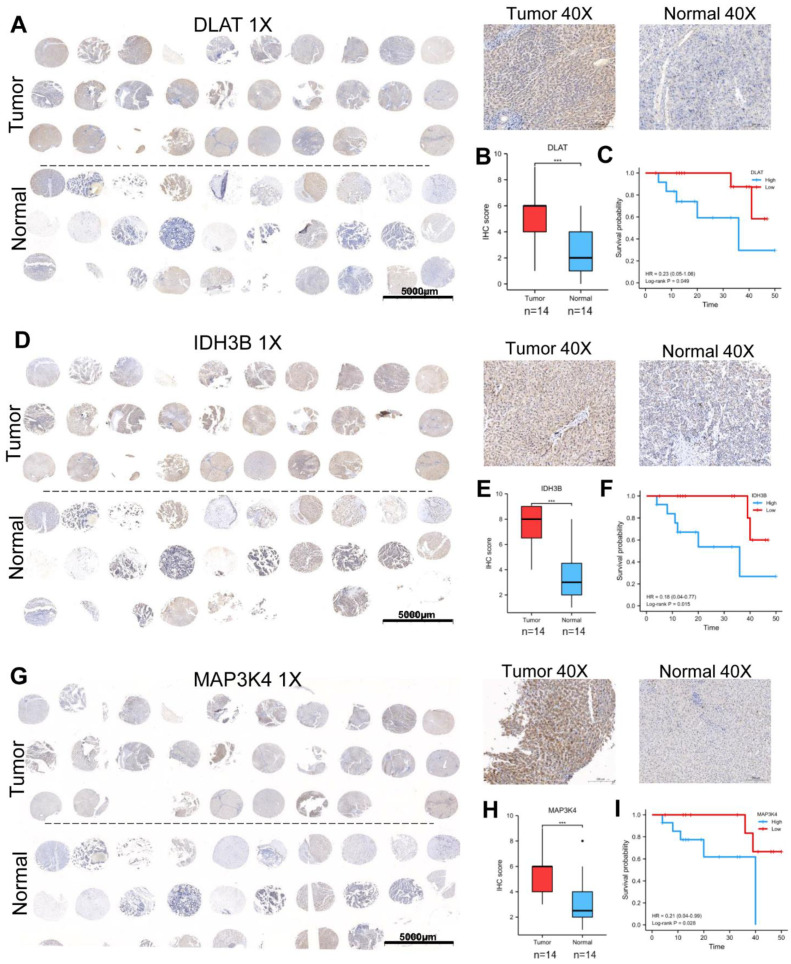
Validation of the expression and prognosis in a real-world HCC cohort. (**A**–**C**) The expression level and prognostic value of DLAT were analyzed by IHC and survival curve. (**D**–**F**) The expression level and prognostic value of IDH3B were analyzed by IHC and survival curve. (**G**–**I**) The expression level and prognostic value of MAP3K4 were analyzed by IHC and survival curve. *** *p* < 0.001.

We collected 30 pairs of HCC tissues and normal liver tissues to make the tissue microarray (Table 1). Excluding sample loss during tissue microarray fabrication and staining, 28 pairs of samples are available. The protein expression levels of DLAT, IDH3B, and MAP3K4 were detected by IHC. The high/low expression of the target protein was relative, defined by the median IHC staining score. Consistent with the previous analysis, our results showed that DLAT presented a high expression level, and the patients with high DLAT had poorer OS (Log-rank *p* = 0.049) (Figure 6A–C). Similar results were found in IDH3B (Log-rank *p* = 0.015) (Figure 6D–F) and MAP3K4 (Log-rank *p* = 0.028) (Figure 6G–I).

### 3.6. Prediction of the Upstream TFs in HCC

**Figure 7:**
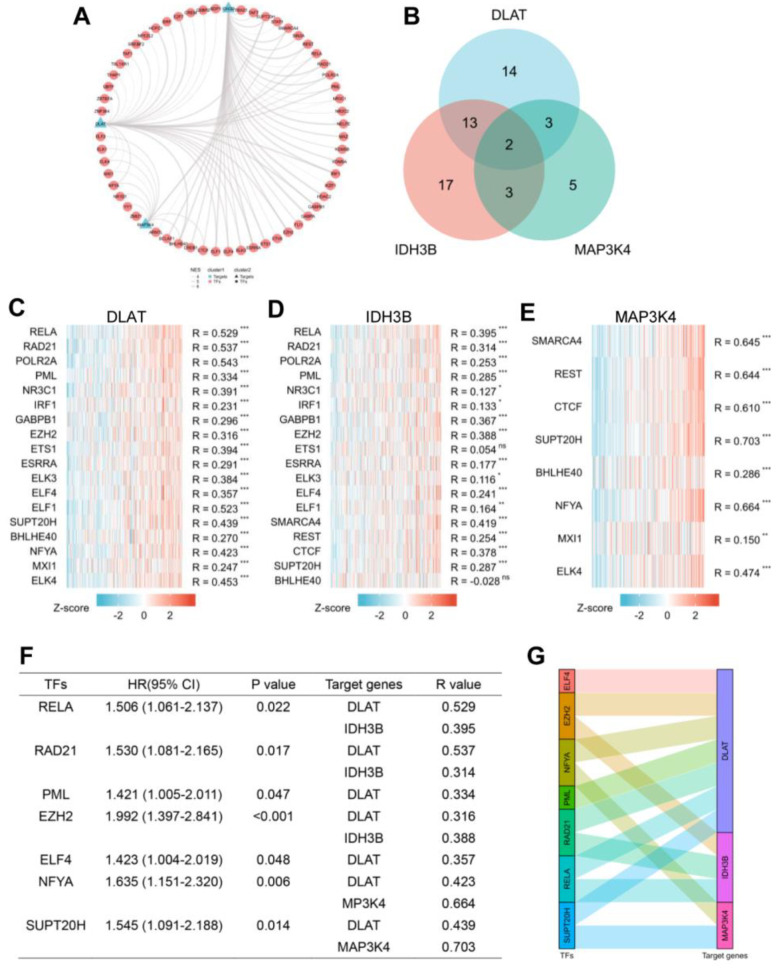
Prediction of potential TF-gene interactions. (**A**) The transcriptional network for DLAT, IDH3B, and MAP3K4. (**B**) Screening for TFs regulating at least 2 genes. (**C**–**E**) The heatmaps of correlations between predicted TFs and target genes. (**F**) Prognostic analysis of TFs with spearman R > 0.3. (**G**) The Sankey diagram showed the corresponding relationships between TFs and target genes. * *p* < 0.05, ** *p* < 0.01, *** *p* < 0.001 and ns, no significance.

To better understand the regulatory mechanisms, transcriptome analysis was performed using GRNdb in HCC. First, we predicted 32 TFs targeted DLAT, 35 TFs targeted IDH3B, and 14 TFs targeted MAP3K4 (Table S2) and constructed a transcriptional regulatory network (Figure 7A). To further explore the intrinsic relationship of the gene set, we screened TFs targeting at least two genes for subsequent analysis (Figure 7B). The correlation between TFs and target genes was shown by heatmaps (Figure 7C–E). Based on correlation analysis (R > 0.3) and prognostic analysis (*p* < 0.05), we finally identified seven potential TFs: RELA, RAD21, PML, EZH2, ELF4, NFYA, and SUPT20H (Figure 7F). The corresponding relationships between TFs and target genes are presented in Figure 7G.

### 3.7. Construction of ceRNA Network in HCC

**Figure 8:**
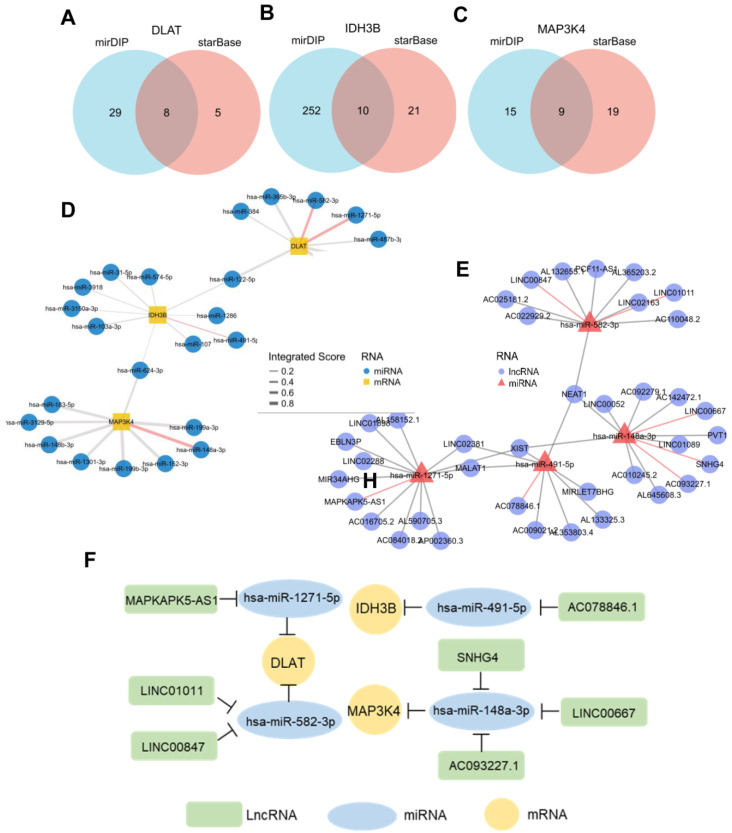
Prediction of potential ceRNA network. (**A**–**C**) Venn diagram found 8 potential miRNAs of DLAT, 10 potential miRNAs of IDH3B, and 9 potential miRNAs of MAP3K4. (**D**) Network for miRNA–mRNA interactions. Red edges represent the miRNAs with prognostic values in TCGA-LIHC. (**E**) Network for miRNA–lncRNA interactions. Red edges represent the miRNAs with prognostic values in TCGA-LIHC. (**F**) The potential mRNA–miRNA–lncRNA competing endogenous RNA (ceRNA) triple regulatory network associated with the prognosis of HCC.

In recent years, more and more evidence has revealed the role of mRNA–miRNA–lncRNA networks in a variety of human cancers. The predicted miRNAs and lncRNAs are from mirDIP and StarBase V2.0. These two databases combined nearly 30 different sources, and we selected the targets with the highest score class for further analysis. Finally, we identified 8 miRNAs that targeted DLAT, 10 miRNAs that targeted IDH3B, and 9 miRNAs that targeted MAP3K4 (Figure 8A–D, Table S3). Based on the classical inverse relationship between miRNAs and target genes, we hypothesized that the upstream miRNAs should theoretically display lower expression levels and favorable prognosis. Therefore, we further evaluated the expression and prognosis of 27 predicted miRNAs in HCC. The results showed that four miRNAs (hsa-miR-1271-5p, hsa-miR-582-3p, hsa-miR-491-5p, and hsa-miR-148a-3p) out of 27 miRNAs functioned as significant prognostic biomarkers for patients with HCC (Figure S2A).

Next, we further predicted those lncRNAs that can potentially bind to the four key miRNAs (Figure 8E, Table S4) based on the StarBase. There is a negative correlation between lncRNAs and miRNAs, according to the ceRNA hypothesis. Combining the expression analysis and survival analysis results for these predicted lncRNAs, we defined the seven lncRNAs: LINC00847 and LINC01011 targeted hsa-miR-582-3p; MAPKAPK5-AS1 targeted hsa-miR-1271-5p; AC078846.1 targeted hsa-miR-491-5p; and SNHG4, LINC00667, and AC093227.1 targeted hsa-miR-148a-3p (Figure S2B). Through the above-integrated analysis, we have constructed the mRNA–miRNA–lncRNA triple network (Figure 8F), which was significantly associated with the prognosis of HCC. The network may also be developed as promising prognostic biomarkers or therapeutic targets for HCC in the future.

### 3.8. The Expression and Mutation Profile in Pan-Cancer

**Figure 9:**
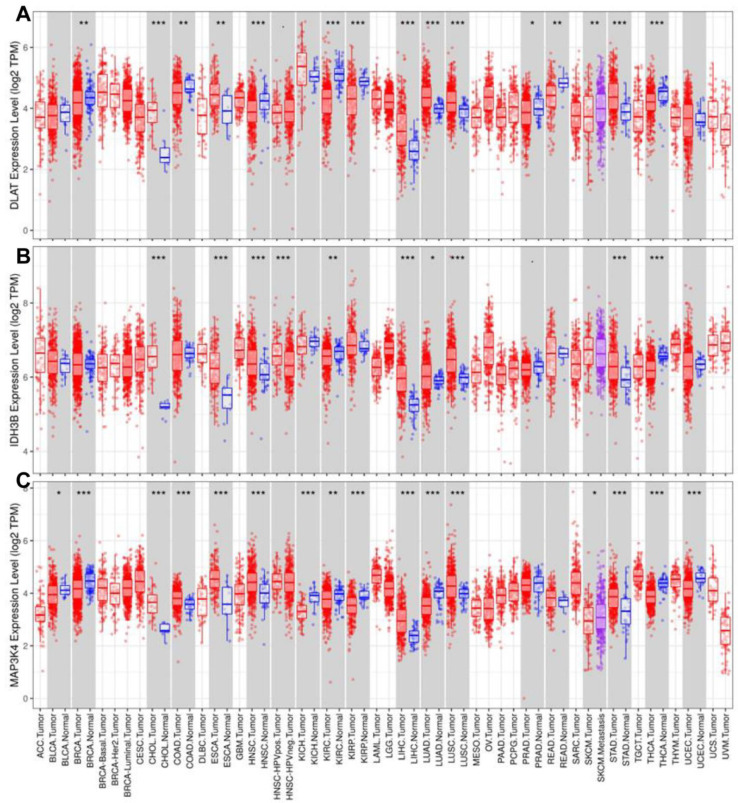
Analysis of expression levels in pan-cancer. The expression levels of DLAT (**A**), IDH3B (**B**), and MAP3K4 (**C**). * *p* < 0.05, ** *p* < 0.01, *** *p* < 0.001.

To further explore the role of the gene set (DLAT, IDH3B, and MAP3K4) across diverse cancer types, we analyzed their expression levels and mutant profiles. We found that the gene set had consistently high expression levels in CHOL, ESCA, LIHC, LUAD, LUSC, and STAD but low expression levels in KIRC and THCA (Figure 9A–C).

**Figure 10:**
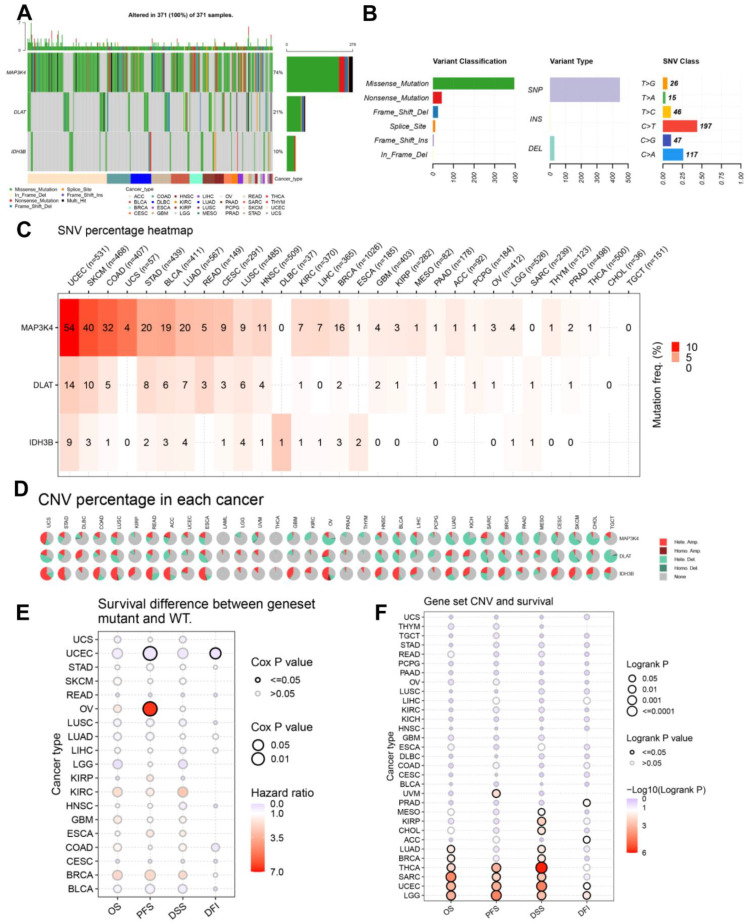
The landscapes of mutation based on GSCA. (**A**) SNV information (deleterious) of MAP3K4, DLAT, and IDH3B in each sample was shown in the waterfall plot. Different mutation types were identified by color. (**B**) According to classification categories, missense mutation, SNP, and C > T mutation accounted for a larger proportion. (**C**) The SNV percentage heatmap of MAP3K4, DLAT, and IDH3B in pan-cancer. (**D**) The CNV percentage pie plots of MAP3K4, DLAT, and IDH3B in pan-cancer. (**E**) The survival difference between gene set mutant (deleterious) and wide type in pan-cancer. (**F**) The survival difference between gene set CNV and wide type in pan-cancer. SNP, single-nucleotide polymorphism; SNV, single-nucleotide variant; CNV, copy number variation; OS, overall survival; PFS, progression-free survival; DSS, disease specific survival; DFI, disease free interval.

Mutation data of DLAT, IDH3B, and MAP3K4 were analyzed and visualized in the GSCA online tool. The landscape of mutation in each cancer sample was presented in the waterfall plot (Figure 10A). By comparison, missense mutations, SNPs, and C > T accounted for the largest fraction of SNV in pan-cancer (Figure 10B). SNV heatmap indicated that the mutation rate of MAP3K4 was the highest, and uterine corpus endometrial carcinoma (UCEC) had the highest mutation rate (Figure 10C, Table S5). On the other hand, CNV percentages in pan-cancer are shown in Figure 10D. As for DLAT, the highest copy number amplification (>33%) was found in DLBC, and the highest copy number deletion (>81%) was found in TGCT. As for IDH3B, the highest copy number amplification (>62%) was found in UCS, and the highest copy number deletion (>29%) was found in READ. As for MAP3K4, the highest copy number amplification (>44%) was found in UCS, and the highest copy number deletion (>77%) was found in KICH (Table S5).

In addition, the prognostic value of the gene set mutations was also analyzed in pan-cancer (Figure 10E,F). The endpoints of OS, PFI, DFI, and DSS data are in accordance with Liu’s suggestion [25]. The results suggested that the gene set mutant had better PFS and DFI than the wild type in UCEC but had worse PFS in OV. Moreover, the gene set amplification/deletion type in THCA, SARC, UCEC, and LGG had consistently better OS, PFS, and DSS than the wild type.

### 3.9. Drugs Sensitivity and Response Analysis

**Figure 11:**
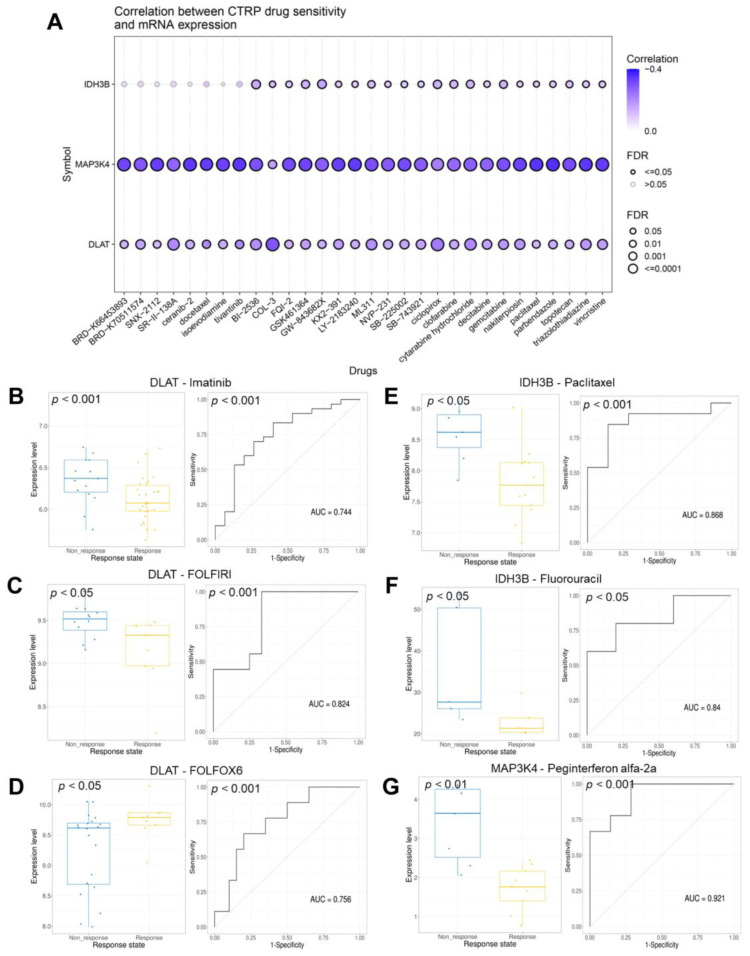
Analysis of the drug sensitivity and response. (**A**) The bubble plot showed the correlations between genes and drugs (top 30). The drugs were ranked by the integrated level of correlation coefficient and FDR of searched genes. (**B**–**D**) The ability of DLAT to predict response to imatinib, FOLFIRI, and FOLFOX6 therapeutic regimen. (**E**–**F**) The ability of IDH3B to predict response to paclitaxel and fluorouracil. (**G**) The ability of MAP3K4 to predict response to peginterferon alfa-2a. FOLFIRI, Fluorouracil + Irinotecan + Leucovorin; FOLFOX6, Fluorouracil + Leucovorin + Oxaliplatin.

The correlations between the gene set expression and drug sensitivity (top 30) in pan-cancer were summarized in Figure 11A and Table S6. We found that the expression level of the gene set was generally negatively correlated with drug sensitivity. Next, we evaluated the response of the gene set to therapeutic drugs in six real-world trial cohorts (Table 2). Figure 11B–D revealed the ability of DLAT to predict response to Imatinib, FOLFIRI, and FOLFOX6 regimen were 0.744, 0.824, and 0.756. Figure 11E,F revealed the ability of IDH3B to predict response to Paclitaxel and Fluorouracil were 0.868 and 0.84. Figure 11G revealed the ability of MAP3K4 to predict response to Peginterferon alfa-2a was 0.921.

### 3.10. Correlation Analysis of Immune Cell Infiltration

**Figure 12:**
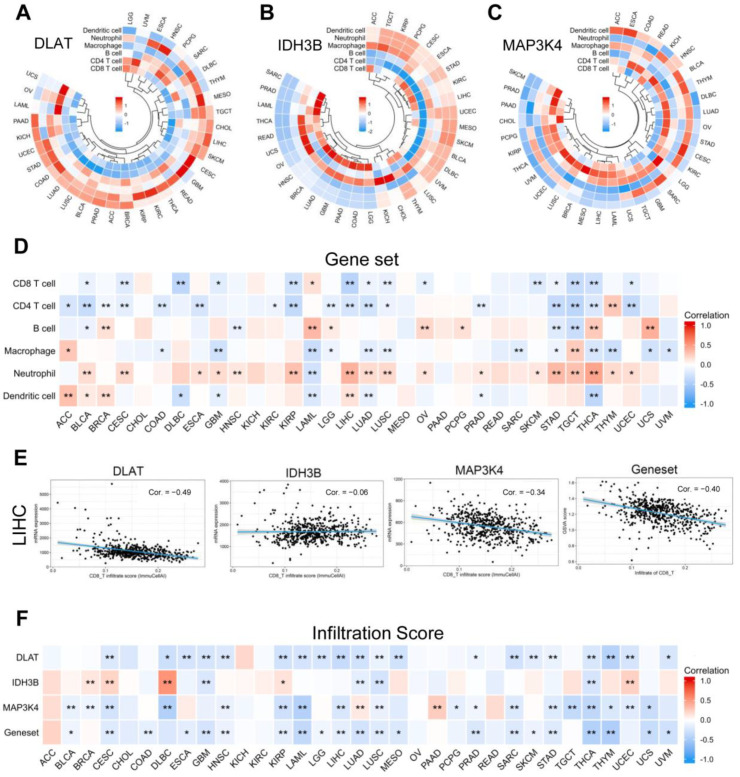
Correlation analysis of immune cell infiltration. (**A**–**C**) The correlation between the single gene expression levels and the infiltration of 6 immune cells. (**D**) The correlation between the gene set expression (based on GSVA score) and the infiltration of 6 immune cells. (**E**) Scatter plots showed that CD8 T cell infiltration was negatively correlated with the expression of DLAT, IDH3B, MAP3K4, and the gene set in TCGA-LIHC. (**F**) The correlation between gene expression levels and infiltration score in pan-cancer. * *p* < 0.05, ** *p* < 0.01.

With the popularity of immunotherapy, attention has been paid to the role of the tumor microenvironment in the process of tumor development, metastasis, recurrence, and drug resistance [26]. We analyzed the relationship between the gene set expression levels and immune cell infiltration in 33 human tumors. As shown in Figure 12A–C, we selected six common immune cells: CD8 T cell, CD4 T cell, B cell, macrophage, neutrophil, and dendritic cell. To show the results more clearly as a whole, we used the GSVA score to replace the gene set expression value (Figure 12D) (Table S7). GSVA score represented the comprehensive level of gene set expression and was positively correlated with gene set expression [27]. It is generally accepted that CD8 T cells are the main performers of the immune response. Specifically in LIHC, we found that DLAT, MAP3K4, and CD8 T cell infiltration were negatively correlated (Figure 12E). Further evaluation by the infiltration score results suggested that the expression level of the gene set was inversely correlated with immune cell infiltration in general (Figure 12F).

## 4. Discussion

In recent decades, NASH has gradually developed into the most common chronic liver disease, which is closely associated with obesity, type 2 diabetes mellitus, dyslipidemia, and hypertension [28]. NASH-driven HCC is a serious public health concern as the number of cases increases year by year. Current international clinical practice guidelines for HCC do not consider etiology. Therefore, the explorations of NASH-related biomarkers and prognostic models may help stratify patients and predict prognosis, and reveal potential therapeutic targets.

Studies of the mechanisms of NASH-driven HCC have provided us with some potential targets. STARD1 is considered to affect the progression of HCC by promoting the synthesis of primary bile acids through the mitochondrial pathway [29]. Activation of the E2F1–E2F2–CPT2 axis established a lipid-rich pro-tumor environment [30]. FGF21, which controls inflammation and anti-fibrosis, has been identified as a novel intervention target for NASH–HCC transformation [31]. Targeted inhibition of the LPL/FABP4/CPT1 axis can block malignant transformation during NASH [32]. Studies of senescent cells also indicated that nicotinamide adenine dinucleotide (NAD), silence information regulator protein family (sirtuins), and mechanistic target of rapamycin (mTOR) were the major signaling pathways involved in NASH-driven HCC [33]. Augmenter of liver regeneration (ALR) is an important enzyme to maintain mitochondrial dynamics [34]. Zheng et al. proved that the ubiquitination degradation of ALR had a potential contribution to NASH-driven HCC progression at the mitochondrial level [35].

We defined three common DEGs between NASH and HCC. DLAT, or dihydrolipoamide S-acetyltransferase, has been reported to mediate glycolysis reprogramming in non-small cell lung cancer [36]. It is also considered to be one of the key proteins regulating cuproptosis [37]. IDH3B (isocitrate dehydrogenase 3β) is thought to cooperate with the cell cycle and participate in cell proliferation in squamous esophageal carcinoma [38]. MAP3K4 (mitogen-activated protein kinase kinase kinase 4), as an important member of the MAPK pathway, is known to be a regulatory gene related to abnormal lipid droplet accumulation [39]. However, their roles in HCC have not been reported.

We constructed a prognostic risk model using these three DEGs through LASSO regression analysis and univariate Cox analysis. Transcriptome and ceRNA network analyses were performed to better understand the regulatory mechanisms. We finally identified seven potential TFs that may be involved in the upstream regulation of this gene set. In addition, we constructed a prognosis-related ceRNA network consisting of seven lncRNAs, four miRNAs, and three mRNAs. The network may also be developed as promising prognostic biomarkers or therapeutic targets for HCC in the future. The mutant profile and drug sensitivity/response analysis further guided gene diagnosis and clinical medication. It is well known that the treatment of HCC is a comprehensive treatment strategy based on surgery. There are few drugs for HCC compared to other cancers. Therefore, the hypothesis and exploration of “drug functional relocation” and “drug combination” have become more important and urgent for HCC. Although none of these drugs were used in the treatment of HCC, this study at least provided novel therapeutic hypotheses and potential therapeutic targets.

It is well known that immunotherapy for advanced HCC has shown promising results in animal models and clinical trials. However, surprisingly, NASH–HCC mouse models did not respond to PD-1/PD-L1 treatment but instead exacerbated liver fibrosis and increased the incidence of tumors [40]. Dominik Pfister et al. analyzed three clinical trials (CheckMate 459, IMbrave 150, and Keymat-240) and found that immunotherapy did not improve survival in patients with non-viral HCC [41,42,43,44]. Our study showed that the infiltration of immune cells, mainly CD8 T cells, was negatively correlated with the gene set expression. We preliminarily believe that DLAT and MAP3K4 are one of the targets, and interfering with their expression may improve the immunotherapy efficacy of NASH–HCC.

However, our study has some limitations. First, further exploration of its biological functions and mechanisms in vivo and in vitro is necessary, which we are carrying out. Secondly, single-cell sequencing and flow sorting are needed to further verify the correlation between the gene set and the tumor immune microenvironment. Thirdly, the implementation of an international multi-regional clinical trial is necessary to avoid selection bias.

## 5. Conclusions

To sum up, by combining machine learning with multi-omics analysis, we found a set of NASH-related prognostic biomarkers, which may contribute to the development of more personalized and precise treatment strategies for HCC.

## Figures and Tables

**Table 1:** Clinical information of HCC patients from Xiangya Hospital.

Characteristic	Levels	Overall
Age	>60	16 (53.3%)
	≤60	14 (46.7%)
Gender	Female	3 (10%)
	Male	27 (90%)
Pathological stage	I	11 (36.7%)
	II	11 (36.7%)
	III	5 (16.7%)
	IV	3 (10%)
T stage	T1	10 (33.3%)
	T2	13 (43.3%)
	T3	6 (20%)
	T4	1 (3.3%)
N stage	N0	28 (93.3%)
	N1	2 (6.7%)
M stage	M0	29 (96.7%)
	M1	1 (3.3%)
Living status	Alive	23 (76.7%)
	Dead	7 (23.3%)

**Table 2:** Data of drug response from CTR-DB.

Gene	CTR-DB ID	Cancer Type	Therapeutic Regimen	Sample
DLAT	CTR_Microarray_14	Leukemia	Imatinib	45
	CTR_Microarray_15	Colorectal cancer	FOLFIRI	21
	CTR_Microarray_35	Colorectal cancer	FOLFOX6	29
IDH3B	CTR_Microarray_71	Ovarian cancer	Paclitaxel	20
	CTR_RNAseq_410	Stomach cancer	Fluorouracil	10
MAP3K4	CTR_RNAseq_386	Skin cancer	Peginterferon alfa-2a	16

FOLFIRI, Fluorouracil + Irinotecan + Leucovorin; FOLFOX6, Fluorouracil + Leucovorin + Oxaliplatin.

## Data Availability

All original data were available on https://doi.org/10.6084/m9.figshare.21707678.v1, accessed on 11 December 2022. All web links were described in the “Methods” section. Further inquiries can be directed to the corresponding authors.

## Supplementary Materials

The following supporting information can be downloaded at: https://www.mdpi.com/article/10.3390/jcm12041286/s1, Figure S1. Prognostic value of DLAT, IDH3B, and MAP3K4 in patients with Child-Pugh grade B&C; Figure S2. The expression levels and prognostic values of related miRNAs and lncRNAs; Table S1. Protein–protein interaction; Table S2. Predictive transcription factors from GRNdb; Table S3. Predictive miRNAs from mirDIP and StarBase V2.0.; Table S4. Predictive lncRNAs from StarBase V2.0.; Table S5. The landscape of mutation in pan-cancer; Table S6. Drug sensitivity data in pan-cancer; Table S7. Immune infiltration analysis in pan-cancer; Table S8. Abbreviation list.
