# Supplementary material for: A Multi-Omics Analysis of NASH-Related Prognostic Biomarkers Associated with Drug Sensitivity and Immune Infiltration in Hepatocellular Carcinoma
Source: J Clin Med. 2023 Feb 6;12(4):1286. doi: 10.3390/jcm12041286 (PMC9963320; doi:10.3390/jcm12041286)
Supplement: Supplementary file 1 [file jcm-12-01286-s001.zip › Table S8 .pdf]

Abbreviation	Full Name
ALR	augmenter of liver regeneration
BP	biological process
CC	cellular component
ceRNA	competing endogenous RNA
CNV	copy number variation
DEG	differentially expressed gene
DLAT	dihydrolipoamide S-acetyltransferase
GO	Gene Ontology
HCC	hepatocellular carcinoma
IDH3B	isocitrate dehydrogenase 3β
IHC	immunohistochemistry
KEGG	Kyoto Encyclopedia of Genes and Genomes
LASSO	least absolute shrinkage and selection operator
LncRNA	long non-coding RNA
MAP3K4	mitogen-activated protein kinase kinase kinase 4
MF	molecular function
mTOR	mechanistic target of rapamycin
miRNA	microRNA
NAD	nicotinamide adenine dinucleotide
NASH	nonalcoholic steatohepatitis
OS	overall survival
PPIs	protein-protein interactions
ROC	receiver operating characteristic
sirtuins	silence information regulator protein family
SNV	single-nucleotide variant
TF	transcription factor
