# Supplementary figures and images for: A Multi-Omics Analysis of NASH-Related Prognostic Biomarkers Associated with Drug Sensitivity and Immune Infiltration in Hepatocellular Carcinoma
Source: J Clin Med. 2023 Feb 6;12(4):1286. doi: 10.3390/jcm12041286 (PMC9963320; doi:10.3390/jcm12041286)

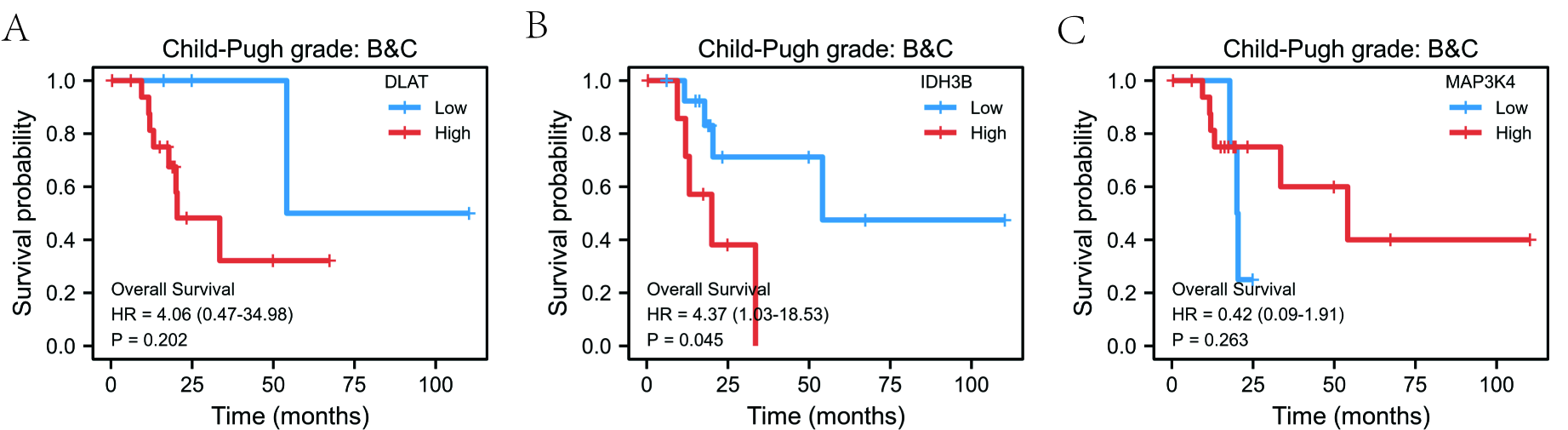

Supplement: Supplementary file 1 [file jcm-12-01286-s001.zip › Figure S1.tif]

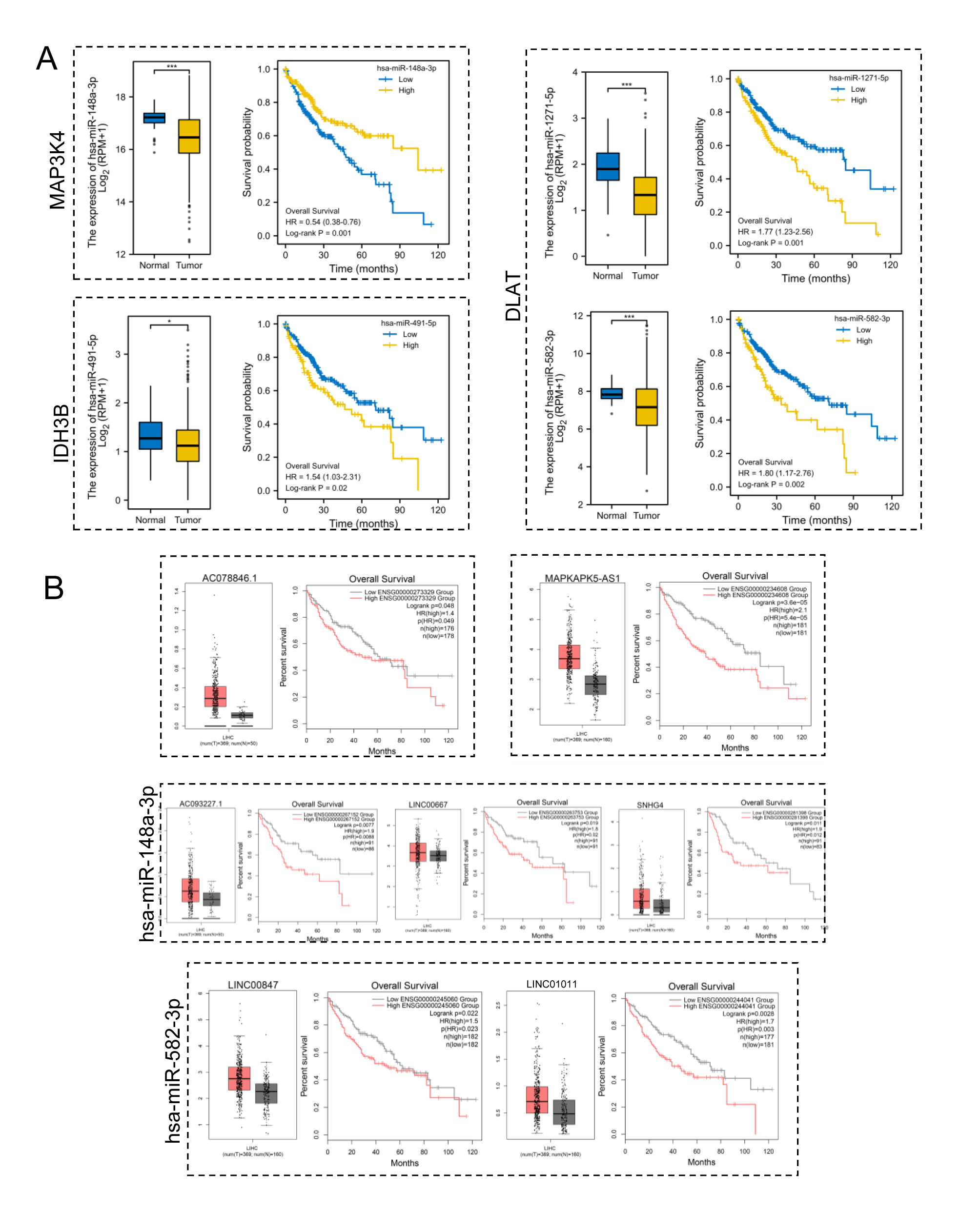

Supplement: Supplementary file 1 [file jcm-12-01286-s001.zip › Figure S2 .tif]
